# Attachment Style and Perinatal Depressive Symptoms Across the Perinatal Period in Japan

**DOI:** 10.3390/children13030332

**Published:** 2026-02-26

**Authors:** Michiko Oyamada, Mitsue Sato, Tatsuma Nakao, Masahiro Sugimoto

**Affiliations:** 1 Faculty of Health Care and Nursing, Juntendo University, Mishima 411-8787, Shizuoka, Japan; m.oyamada.dl@juntendo.ac.jp; 2 Department of Nursing, Kanagawa Dental University Junior College, Yokosuka 238-8580, Kanagawa, Japan; sato.mitsue@kdu.ac.jp; 3 Faculty of Education, University of the Ryukyus, Nishihara 903-0213, Okinawa, Japan; tatsuma@cs.u-ryukyu.ac.jp; 4 Institute for Advanced Biosciences, Keio University, Tsuruoka 997-0052, Yamagata, Japan; 5 Institute of Medical Science, Tokyo Medical University, Shinjuku, Tokyo 160-8402, Japan

**Keywords:** postpartum depression, attachment style, cortisol

## Abstract

**Highlights:**

**What are the main findings?**

- Attachment insecurity was associated with elevated depressive symptoms across the perinatal period.
- Higher psychological stress responses were consistently linked to EPDS positivity.
- Salivary cortisol showed no consistent association with depressive symptoms.

**What is the implication of the main findings?**

- Brief psychosocial screening during pregnancy may help identify women at risk for perinatal depression.

**Abstract:**

**Background/Objectives:** Perinatal depressive symptoms are influenced by psychosocial and relational factors. This study examined stage-specific associations between adult attachment style, psychological stress responses, satisfaction with the childcare environment, and depressive symptoms across five perinatal stages in Japan. **Methods:** This repeated cross-sectional study included 417 independent assessment datasets collected during the first, second, and third trimesters, and at two weeks and one month postpartum. Depressive symptoms were assessed using the Edinburgh Postnatal Depression Scale (EPDS). Adult attachment was measured using the Relationship Questionnaire, and psychological stress responses were measured using the Stress Response Scale-18 (SRS-18). Salivary cortisol was analyzed in a subset of participants. **Results:** Elevated depressive symptoms (EPDS+) were observed in approximately 10–15% of participants across stages. Attachment insecurity was associated with higher odds of EPDS+ at one month postpartum (OR 12.1, 95% CI 1.35–109). Higher SRS-18 scores were consistently associated with increased odds of EPDS+ across stages (e.g., OR 20.9, 95% CI 5.46–80.0 in the second trimester). Lower satisfaction with the childcare environment was associated with elevated depressive symptoms during pregnancy. No consistent association was observed between salivary cortisol and EPDS+. **Conclusions:** Adult attachment insecurity and psychological stress responses were associated with perinatal depressive symptoms across stages. By clarifying stage-specific psychosocial patterns, these findings support stress–attachment frameworks, suggesting that attachment insecurity may heighten vulnerability during the perinatal transition, provide culturally specific evidence from Japan, and underscore the potential value of brief psychosocial screening in routine perinatal care.

## 1. Introduction

Postpartum depression (PPD) is a significant mental health problem affecting approximately 13–19% of new mothers worldwide [1,2]. PPD has profound effects on both mothers and infants, leading to impaired mother-infant bonding, difficulties with breastfeeding, and long-term developmental problems in children [3,4]. Furthermore, in the most severe cases, it can lead to maternal suicide, which is a leading cause of maternal mortality in the postpartum period [5,6]. In Japan, the prevalence of PPD is reported to be around 10% [7,8], indicating a serious maternal mental health problem. In Japan, there is an increasing trend of suicide among women within the first year postpartum [9], highlighting the importance of early identification and intervention to mitigate these adverse outcomes.

The Edinburgh Postnatal Depression Scale (EPDS) is a widely used self-report questionnaire for screening PPD [10]. The EPDS has demonstrated reliability and validity in diverse populations and is considered a widely used and well-validated screening instrument for postpartum depression [11]. In addition, studies have examined antenatal EPDS scores and pregnant women’s background factors to predict early PPD [12,13]. These studies have identified risk factors for postpartum depression and indicated the potential for preventive interventions. However, while the EPDS provides valuable insight into the psychological aspects of PPD, it does not capture the biological basis.

Recent research has explored the role of biological markers in diagnosing and understanding PPD. Cortisol, a hormone released in response to stress, is primarily measured in biological samples such as blood, urine, and saliva. In particular, cortisol has been identified as a potential biomarker for PPD [14]. Salivary cortisol measurement is a non-invasive and convenient method for assessing hypothalamic–pituitary–adrenal (HPA) axis activity [15]. Studies investigating the relationship between salivary cortisol and PPD have reported that women with postpartum depression have abnormal cortisol secretion patterns [16,17]. Furthermore, elevated cortisol levels are associated with both antenatal and postnatal depression, suggesting a link between stress and the onset of depressive symptoms in new mothers [18,19]. These studies indicate that salivary cortisol measurements may provide valuable insights into the physiological stress response of postpartum women and may be helpful for the early detection and intervention of PPD.

Attachment theory, proposed by John Bowlby (1969), posits that the emotional bond formed between infants and their primary caregivers plays a crucial role in subsequent social and emotional development [20]. Bowlby theorized that attachment serves as a secure base, enabling children to explore their surroundings and cope with stress with confidence. Mary Ainsworth further advanced this theory by employing the “Strange Situation” experimental procedure to categorize children’s attachment styles as “secure,” “insecure–avoidant,” or “insecure–ambivalent” [21]. Although these classifications were originally developed for infants, similar conceptual frameworks have been applied to adult attachment patterns. PPD has been demonstrated to exert an influence on maternal attachment styles, with the potential to exert adverse effects on mother-infant interactions and child development [22]. Research findings indicate that insecure attachment styles may serve as risk factors for the development of PPD [23]. Consequently, it is imperative to incorporate the assessment of maternal attachment styles into postpartum mental health evaluations to facilitate the timely identification and management of PPD.

International studies conducted in Europe and North America have similarly reported associations between attachment insecurity and perinatal depressive symptoms [24,25], suggesting that attachment-related vulnerability may represent a cross-cultural risk factor. Prospective and longitudinal research has further demonstrated that attachment insecurity may moderate the impact of perinatal stress on depressive outcomes [25]. However, the strength and expression of these associations may vary across sociocultural contexts, including family structures, partner support, and expectations regarding maternal roles [2]. Cross-national reviews have emphasized that social support systems and culturally embedded parenting norms influence the prevalence and manifestation of perinatal depression [26]. In East Asian contexts such as Japan, where extended family involvement and social norms surrounding motherhood differ from Western settings, the psychosocial dynamics of perinatal mental health may follow distinct patterns [27]. Therefore, culturally specific empirical evidence is needed to clarify whether attachment-related vulnerabilities operate similarly across contexts.

Therefore, the present study aimed to examine the cross-sectional associations between perinatal depressive symptoms and psychosocial and relational factors, including adult attachment style, psychological stress responses, and satisfaction with the childcare environment, across multiple perinatal stages in Japan. In addition, salivary cortisol was assessed as an exploratory physiological stress marker to complement psychological measures. We hypothesized that attachment insecurity, higher psychological stress responses, and lower satisfaction with the childcare environment would be associated with elevated perinatal depressive symptoms at each perinatal stage. In contrast, associations with salivary cortisol would be less consistent. To address these aims, we conducted a repeated cross-sectional study in which independent samples of women were assessed at five perinatal stages, from early pregnancy to the early postpartum period. By focusing on stage-specific associations rather than within-individual change, this study seeks to clarify patterns of psychosocial vulnerability related to perinatal depressive symptoms while avoiding causal or longitudinal assumptions.

## 2. Materials and Methods

### 2.1. Study Design

This study employed a repeated cross-sectional observational design, analyzing data collected at five separate time points as independent snapshots, without modeling within-subject changes over time. The five time points included the first trimester, the second trimester, the third trimester, two weeks postpartum, and one month postpartum. Because participant identifiers could not be consistently linked across all time points, longitudinal modeling was not feasible, and each assessment was treated as an independent cross-sectional snapshot. At each of the five perinatal stages, data were collected from different individuals. No participant contributed data at more than one point. Accordingly, all observations were independent, and each stage represents a distinct cross-sectional sample.

### 2.2. Participants and Recruitment

**Figure 1:**
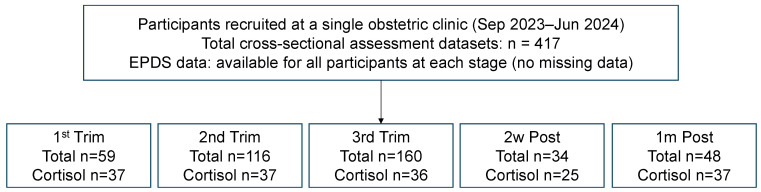
Participant flows across five perinatal stages and the availability of salivary cortisol samples. A total of 417 independent cross-sectional assessment datasets were collected at five predefined perinatal stages (first trimester, second trimester, third trimester, two weeks postpartum, and one month postpartum). EPDS data were available for all participants at each stage. Salivary cortisol samples were obtained in a subset of participants depending on feasibility and consent. All stages represent independent cross-sectional samples, and no participant contributed data at more than one stage.

Participants were recruited from a single obstetric clinic in Japan between September 2023 and June 2024. Inclusion criteria were age ≥18 years, singleton pregnancy, and ability to complete self-administered questionnaires in Japanese. Women with known psychiatric diagnoses or pregnancy complications were excluded. Data were collected at five predefined perinatal stages (first, second, and third trimesters of pregnancy; two weeks postpartum; and one month postpartum). At each stage, participants were recruited independently during routine prenatal or postpartum visits. Each assessment represented a different individual at a single perinatal stage, and no participant contributed data at more than one assessment point. All questionnaire data were therefore analyzed as independent cross-sectional samples without linkage across stages. Salivary samples were collected at the same visits when feasible. Among all participants included in the study, 172 individuals provided saliva samples for cortisol analysis. Because saliva collection depended on visit timing, participant consent, and logistical feasibility, biological data were available only for a subset of the cross-sectional samples. Accordingly, analyses involving salivary cortisol were conducted as exploratory, stage-specific cross-sectional analyses (Figure 1). This study was conducted as an observational investigation. No structured psychological or behavioral interventions were implemented as part of the study protocol. Participants received routine obstetric care only during pregnancy and the postpartum period.

### 2.3. Psychological Assessments

Perinatal depressive symptoms were assessed using the Edinburgh Postnatal Depression Scale (EPDS), a 10-item self-report questionnaire validated for use in Japanese populations. Stress responses were measured using the Stress Response Scale-18 (SRS-18), which evaluates psychological stress across multiple domains. Adult attachment style was assessed using the Relationship Questionnaire (RQ), a validated self-report measure that categorizes individuals into four attachment styles: secure, fearful, preoccupied, and dismissing.

The attachment styles were also reclassified into two broader categories for secondary analyses. Participants were dichotomized into secure and insecure groups to enhance statistical power.

The collected information included the following variables: (1) age, (2) parity, (3) gestational age, (4) history of stillbirth or miscarriage, (5) psychiatric history, (6) social support (from husband, biological mother, and other sources), (7) economic concerns, (8) living environment, (9) attachment style, (10) SRS-18 score, and (11) EPDS score.

For the EPDS, a cut-off score of ≥11 was used to indicate potential depression during pregnancy [28,29,30], whereas a cut-off score of ≥9 was applied in the postpartum period [11,31]. These different thresholds were selected based on validation studies specific to antenatal and postpartum populations. Attachment styles were assessed and categorized into four types: secure, dismissing, preoccupied, and fearful. Attachment style was also dichotomized for the analyses into secure versus insecure categories. For the SRS-18, a score ≥18 indicated a high stress level, whereas a score <18 indicated a low stress level [32].

Adult attachment styles were assessed using the Relationship Questionnaire (RQ), which presents four brief descriptions corresponding to secure, fearful, preoccupied, and dismissing attachment styles. Participants rated the extent to which each description applied to them on a 7-point Likert scale.

For analytical purposes, attachment styles were classified in two ways. First, participants were categorized into four styles—secure, fearful, preoccupied, and dismissing—based on their forced-choice responses on the RQ. Second, attachment styles were dichotomized into secure and insecure groups for secondary analyses to increase statistical power.

### 2.4. Biological Data

Salivary cortisol analyses were exploratory due to the limited sample size and incomplete pairing across time points. Salivary cortisol was collected as a supplementary biological marker to explore physiological stress responses. Samples were obtained at routine prenatal and postpartum visits using a standardized passive drool method. To ensure homogeneity of sample collection, saliva samples were obtained under standardized conditions within the same clinical facility. Based on preliminary validation of diurnal variation, samples in the main study were collected after breakfast during outpatient visits to minimize circadian variation. Participants were instructed to avoid oral hygiene procedures and strenuous physical activity for at least one hour before the sample collection. All samples were immediately cooled and stored at −80 °C until analysis. Details regarding sampling times, storage procedures, and assay protocols are provided in Appendix A. Salivary cortisol was measured in a subset of participants (*n* = 172) who provided saliva samples at a single perinatal stage.

### 2.5. Statistical Analysis

All analyses were performed separately for each perinatal stage, treating each stage as an independent cross-sectional sample. Within each stage, participants were classified into high- and low-EPDS groups based on predefined cut-off values for the pregnancy and postpartum periods.

Categorical variables were analyzed using Fisher’s exact test (two-sided). For 2 × 2 comparisons, odds ratios (ORs) and 95% confidence intervals (CIs) were calculated directly. For categorical variables with more than two categories, each non-reference category was compared with the predefined reference category using separate 2 × 2 Fisher’s exact tests, and corresponding ORs and 95% CIs were calculated. ORs were not estimated when any cell count was zero, and no continuity correction was applied.

For continuous variables, comparisons between two groups were conducted using the Mann–Whitney U test. When comparisons involved three or more groups, the Kruskal–Wallis test was applied, followed by Dunn’s multiple-comparison test as a post hoc analysis. Because the sample size for salivary cortisol measurements was limited and differed across stages, analyses involving cortisol were exploratory. For these analyses, participants at each stage were dichotomized using the stage-specific median EPDS score, and cortisol concentrations were compared between groups. All statistical analyses were conducted using JMP Pro (version 16.1.0; SAS Institute, Cary, NC, USA) and GraphPad Prism (version 9.2.0; GraphPad Software, La Jolla, CA, USA). Effect sizes are presented as odds ratios (ORs) with 95% confidence intervals to facilitate interpretation of the magnitude of association. Primary analyses focused on associations between EPDS status and adult attachment, psychological stress responses (SRS-18), and satisfaction with the childcare environment. Other stage-specific variables were examined as exploratory secondary analyses. Given the number of comparisons performed, findings from secondary analyses should be interpreted cautiously, and no formal correction for multiple testing was applied.

### 2.6. Ethical Considerations

This study followed the ethical principles of the Helsinki Declaration. Before participating in the study, participants received verbal and written information. It was emphasized that participation in the study, the option to withdraw after consent, and the interpretation of the results would not affect their subsequent medical care. Participants were informed of their right to withdraw from the study at any time. They were told they also had the right to request deletion of their data and that, in the event of publication, measures would be taken to ensure individuals could not be identified. Ethical approval was obtained from the Nihon Institute of Medical Science (No. 2022012, approved on 13 October 2022).

## 3. Results

Because the study employed a repeated cross-sectional design, all stage-specific comparisons reflect differences between independent samples rather than within-individual changes over time. During pregnancy (Table 1), lower satisfaction with the residential environment for child-rearing was associated with increased odds of elevated depressive symptoms, whereas higher satisfaction was associated with reduced odds. Higher psychological stress responses were consistently associated with increased odds of elevated EPDS across pregnancy stages. In the postpartum period (Table 2), attachment insecurity (two-category model) was associated with higher odds of EPDS, reaching statistical significance at one month postpartum. Higher SRS-18 scores were consistently associated with higher EPDS scores across postpartum assessments. Further exploratory results are presented in Appendix B (Table A1).

**Figure 2:**
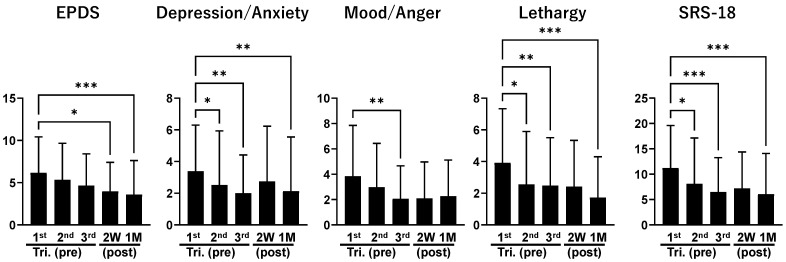
Distributions of scores for the EPDS, SRS-18, and their subscales (Depression/Anxiety, Mood/Anger, and Lethargy) across five perinatal stages. The X-axis corresponds to the first, second, and third trimesters of pregnancy, as well as two weeks and one month postpartum. The Y-axis represents scale scores (no unit). Statistical comparisons were conducted across perinatal stages using the Kruskal–Wallis test with Dunn’s multiple-comparison test. These comparisons reflect differences in score distributions across independent cross-sectional samples rather than within-individual changes over time (* *p* < 0.05, ** *p* < 0.01, *** *p* < 0.001).

Figure 2 presents descriptive distributions of EPDS and SRS-18 scores across the five perinatal stages. Differences in score distributions were observed across stages; however, these findings are descriptive and do not represent within-individual changes over time, given the study’s repeated cross-sectional design. Overall, higher EPDS scores were more frequently observed during pregnancy, whereas lower scores were more common in the postpartum stages. Psychological stress responses, as measured by the SRS-18, showed a similar stage-dependent pattern across assessments.

**Figure 3:**
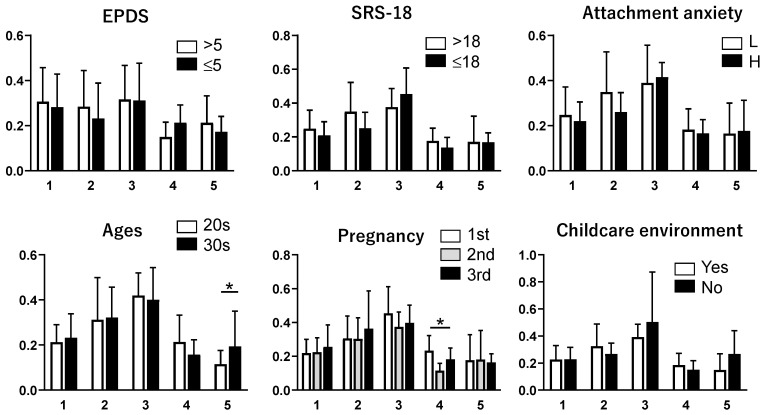
Stage-specific salivary cortisol concentrations across the perinatal period. The X-axis corresponds to the first, second, and third trimesters of pregnancy, as well as two weeks and one month postpartum. The Y-axis shows the salivary cortisol concentrations (μg/dL). Stage-specific sample sizes (*n*) for salivary cortisol were 37, 37, 36, 25, and 37 for the first, second, and third trimesters, two weeks postpartum, and one month postpartum, respectively. EPDS was dichotomized at the sample mean (5): >5 = high; ≤5 = low. Attachment anxiety was dichotomized using the sample mean score (2.6); scores ≥ 2.6 were classified as high (H) and scores < 2.6 as low (L). Statistical comparisons were conducted across perinatal stages using the Kruskal–Wallis test with Dunn’s multiple-comparison test. These comparisons reflect differences in cortisol concentrations across independent cross-sectional samples rather than within-individual changes over time (* *p* < 0.05).

Salivary cortisol analyses were conducted as an exploratory component of the study, informed by a preliminary validation study summarized in Appendix A. As shown in Figure 3, cortisol concentrations varied stage-specifically across pregnancy and the postpartum period. However, no clear or consistent associations were observed between cortisol levels and elevated depressive symptoms (EPDS>5), defined using the sample mean EPDS score as a stage-specific exploratory cut-off, at any assessment stage. Additional subgroup analyses suggested limited stage-specific differences in participant characteristics, but these findings were inconsistent across stages.

## 4. Discussion

This study examined stage-specific associations between perinatal depressive symptoms and psychosocial and physiological factors using a repeated cross-sectional design. Across pregnancy and the early postpartum period, attachment insecurity and higher psychological stress responses were consistently associated with elevated depressive symptoms. These findings align with previous research identifying attachment insecurity as a vulnerability factor for perinatal mood disturbances [24,33]. They are consistent with international reviews emphasizing the importance of psychosocial resilience and relational factors in perinatal mental health [34]. From a theoretical perspective, attachment insecurity may heighten perceived stress and impair emotional regulation, thereby increasing vulnerability to depressive symptoms during the perinatal period [33].

Across both pregnancy and the postpartum period, elevated depressive symptoms were consistently accompanied by higher psychological stress responses, as measured by the SRS-18. This pattern supports prior evidence that psychological stress plays a central role in the development and maintenance of perinatal mood disturbances [27,35]. In this context, the use of validated self-report instruments such as the EPDS and SRS-18 provides a practical and sensitive approach for identifying psychological vulnerability in clinical and public health settings [10,36].

These findings may be interpreted within established attachment and stress–depression frameworks. Attachment theory posits that attachment insecurity is associated with heightened sensitivity to perceived threat and reduced capacity for effective emotional regulation [33]. During the perinatal period, which is characterized by substantial psychological and physiological transitions, individuals with attachment insecurity may experience amplified stress appraisal and diminished coping resources. According to stress–diathesis models, such heightened stress reactivity may increase vulnerability to depressive symptoms when exposed to contextual stressors [37]. The present findings are consistent with this integrative perspective, suggesting that attachment-related emotional regulation patterns and psychological stress responses may jointly contribute to perinatal depressive symptoms.

In contrast, physiological stress markers, including salivary cortisol and chromogranin A (CgA), did not show clear or consistent associations with depressive symptoms or attachment patterns in the present study. This finding differs from some previous reports describing altered cortisol profiles among individuals with depressive symptoms or insecure attachment [15,38]. Several methodological and biological factors may account for this discrepancy. First, cortisol secretion is strongly influenced by diurnal variation, and sampling time can substantially affect measured concentrations [39]. Second, individual variability in stress reactivity and pregnancy-related hormonal changes may obscure group-level differences. Third, single-point cortisol measurements may be insufficient to capture chronic stress exposure or dysregulation of the hypothalamic–pituitary–adrenal (HPA) axis [40]. The absence of clear associations may reflect physiological adaptation of the HPA axis during pregnancy and the postpartum period rather than a true absence of stress-related biological processes.

The selection of salivary cortisol as a physiological stress marker in this study was informed by a preliminary validation experiment examining diurnal variation, summarized in Appendix A. Consistent with previous literature [15,39], this pilot study confirmed a clear circadian rhythm of cortisol, with higher levels in the morning and lower, more variable levels later in the day. In the main study, saliva samples were collected during daytime outpatient visits after breakfast, which may have reduced sensitivity compared with early morning sampling, when cortisol levels are typically higher and more dynamic. While this protocol minimized inter-individual variability and enhanced procedural consistency, it may have limited the ability to detect subtle differences associated with depressive symptoms. Future studies may benefit from standardized morning sampling or repeated measurements to better characterize stress-related endocrine activity.

CgA, a marker of sympathetic nervous system activity, also showed no robust associations with depressive symptoms or attachment patterns. Although CgA has been proposed as a potential biomarker of psychological stress [41,42], its reliability and clinical utility in pregnant and postpartum populations remain uncertain. Overall, these findings suggest that psychological indicators may currently offer greater robustness and clinical relevance than physiological stress markers for assessing perinatal mental health.

Importantly, the cortisol analyses in the present study were conducted in a sample with relatively low overall depressive symptom severity. The average EPDS score among participants included in the cortisol analyses was comparable to levels reported in previous postpartum studies [33]. This restricted range of symptom severity may have further limited the ability to detect clear associations between cortisol concentrations and depressive symptoms.

In addition, the absence of significant associations between salivary cortisol and depressive symptoms may reflect physiological adaptation of the hypothalamic–pituitary–adrenal (HPA) axis during pregnancy and the postpartum period. Pregnancy is characterized by altered baseline endocrine activity and stress responsiveness, which may attenuate detectable differences in cortisol levels between individuals with and without elevated depressive symptoms. Therefore, the present findings should not be interpreted as evidence against biological involvement in perinatal depression, but rather as an indication that single-point salivary cortisol measurements may have limited sensitivity in this context.

The present findings also highlight the potential value of attachment-based screening in perinatal care. Adult attachment styles, as assessed using the Relationship Questionnaire [41], provide insight into interpersonal functioning and emotion regulation. Attachment insecurity may serve as a useful indicator of heightened psychological stress and depressive symptoms, supporting the integration of attachment assessments into routine maternal mental health evaluations [24].

From a clinical perspective, these findings suggest that a brief assessment of adult attachment style and perceived childcare environment satisfaction during pregnancy may help identify women at increased risk for perinatal depressive symptoms. Incorporating psychosocial screening into routine obstetric care may facilitate early identification of vulnerability, particularly among women exhibiting attachment insecurity patterns or elevated psychological stress responses.

Finally, this study has several limitations. These can be broadly categorized as methodological and substantive limitations. Methodological limitations include the repeated cross-sectional design, which precludes causal inference and within-individual analysis. Substantive limitations include limited stage-specific subgroup sizes, potential participant heterogeneity, and the absence of long-term longitudinal follow-up. In addition, residual confounding by unmeasured factors such as socioeconomic conditions, detailed psychiatric history, or partner dynamics cannot be excluded. In addition, a relatively large number of stage-specific comparisons were performed, particularly in exploratory analyses, which increases the possibility of type I error. Therefore, these findings should be interpreted with caution and considered hypothesis-generating rather than confirmatory.

Furthermore, biological stress markers were available only in a subset of participants, which may have reduced statistical power for cortisol-related analyses. Despite these limitations, the present study provides culturally specific data from a Japanese clinical context, offering valuable insight into perinatal mental health and attachment-related processes in a non-Western population. Future longitudinal and multi-site studies across diverse cultural settings are warranted to clarify causal pathways and enhance generalizability.

## 5. Conclusions

In this repeated cross-sectional study, adult attachment insecurity, lower satisfaction with the childcare environment, and higher psychological stress responses were associated with elevated perinatal depressive symptoms across selected perinatal stages. These findings highlight the potential relevance of psychosocial and relational factors in perinatal mental health, while acknowledging that causal relationships cannot be inferred from the present design. Future longitudinal studies with repeated within-individual assessments are needed to clarify causal pathways and long-term maternal and child outcomes. From a clinical perspective, integrating a brief assessment of adult attachment style and psychosocial stress responses into routine perinatal care may facilitate earlier identification of women at increased risk for depressive symptoms and support timely, targeted interventions aimed at promoting maternal mental health.

## Figures and Tables

**Table 1:** Associations between psychosocial factors and elevated depressive symptoms during pregnancy in a repeated cross-sectional study.

Timing	Category	EPDS-	EPDS+	OR	95% CI	*p*
Satisfaction with residential environment for child-rearing						
1st trimester	No	14	5	-	-	-
	Yes	35	5	0.4	0.100–1.60	0.2
2nd trimester	No	13	7	-	-	-
	Yes	89	7	0.146	0.0441–0.484	0.0017 ***
3rd trimester	No	17	4	-	-	-
	Yes	133	6	0.192	0.0491–0.749	0.017 *
SRS-18						
1st trimester	Low	43	4	-	-	-
	High	6	6	10.8	2.34–49.5	0.0023 **
2nd trimester	Low	94	6	-	-	-
	High	6	8	20.9	5.46–80.0	<0.001 ***
3rd trimester	Low	139	6	-	-	-
	High	6	4	15.4	3.42–69.6	<0.001 ***

Elevated depressive symptoms (EPDS+) were defined as an Edinburgh Postnatal Depression Scale (EPDS) score ≥11 and were used as the outcome variable. Categorical variables are presented as binary categories (e.g., Yes/No or Low/High), with the lower or negative category (No or Low) used as the reference. The reference category is indicated by “-“ in the OR and 95% CI columns. * *p* < 0.05, ** *p* < 0.01, and *** *p* < 0.001.

**Table 2:** Associations between psychosocial factors and elevated depressive symptoms in the postpartum period in a repeated cross-sectional study.

Timing	Category	EPDS-	EPDS+	OR	95% CI	*p*
Attachment security (two-category model)						
2 weeks	Secure	17	0	-	-	-
	Insecure	13	4	7.36	0.358–151	0.19
1 month	Secure	29	1	-	-	-
	Insecure	12	5	12.1	1.35–109	0.026 *
SRS-18						
2 weeks	Low	27	2	-	-	-
	High	1	2	27.0	1.65–443	0.035 *
1 month	Low	39	3	-	-	-
	High	2	3	19.5	2.29–166	0.011 *

Elevated depressive symptoms (EPDS+) were defined as an Edinburgh Postnatal Depression Scale (EPDS) score ≥9 and were used as the outcome variable. Categorical variables are presented as binary categories (e.g., Yes/No or Low/High), with the lower or negative category (No or Low) used as the reference. The reference category is indicated by “-“ in the OR and 95% CI columns. * *p* < 0.05.

**Figure A1:**
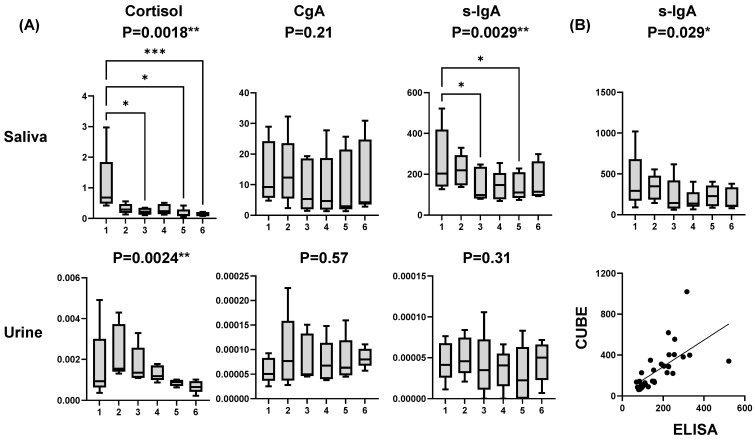
Diurnal validation of biological hormones. (**A**) The ELISA-based absolute values of cortisol, CgA, and s-IgA in saliva (upper) and urine (lower) samples. (**B**) Cube Reader-based salivary s-IgA (upper). The X-axis of the box plots shows saliva collection times: 1 and 2 before breakfast, 3 and 4 before lunch, and 5 and 6 before dinner. The box represents the minimum and maximum values, the quartiles, and the median. The Y-axis of the box plots shows the concentrations in μg/dL (Cortisol), μg/mL (s-IgA), and pmol/mL (CgA). Those of urinary data are divided by the concentration of creatinine (μmol/L). The *p*-value (Friedman test) is shown in each graph. Dunn’s post-test to evaluate differences between the initial measurement and other data points (* *p* < 0.05, ** *p* < 0.01, and *** *p* < 0.001). The lower panel of B) compares salivary s-IgA levels using the CUBE reader and ELISA. The correlation between the quantitative values obtained by the two methods was R = 0.82 (*p* < 0.0001; Spearman’s rank test). The regression line is Y = 1.3 × X + 27.

## Data Availability

The data presented in this study are not publicly available due to privacy and ethical restrictions, as they contain sensitive personal information of the participants.

## Abbreviations

The following abbreviations are used in this manuscript:

EPDS	Edinburgh postnatal depression scale
SRS-18	Stress response scale-18
PPD	postpartum depression
CgA	chromogranin A
s-IgA	secretory immunoglobulin A

## Appendix A

### Appendix A.1. Design of Stress-Related Biomarkers

This study was conducted in two phases. Preliminary study: Saliva and urine samples were collected longitudinally throughout the day to quantify stress-related hormones. Three types of hormones were assessed, and salivary cortisol was selected as a marker of stress level; a saliva collection protocol was defined. Based on preliminary findings showing pronounced diurnal variation before breakfast and more stable concentrations thereafter, saliva samples in the main study were collected between approximately 9:00 and 11:00 AM during routine outpatient visits after breakfast to minimize circadian variability. Primary research: Saliva samples at five time points when questionnaire data were collected, using a cross-sectional study design for quantitative analysis. Pregnant or postpartum women who visited the outpatient clinic were included. Salivary cortisol was quantified and analyzed.

### Appendix A.2. Biomarker Quantification Methods

#### Appendix A.2.1. Sample Collection

Unstimulated saliva samples were collected from outpatients in the Department of Obstetrics and Gynecology at a hospital. Polypropylene tubes with 15 mL polyethylene caps (Falcon) were used for saliva collection. Polypropylene straws with a diameter of 1.1 mm were also used. Participants were instructed to avoid oral hygiene practices and strenuous exercise for 1 h before collection. Unstimulated saliva was collected in a quiet, private room after gently rinsing the mouth, allowing accumulated saliva to drip into the tube through a straw. Samples were collected while cooled with ice. The collection time was approximately 3 min. Saliva samples were immediately stored at −80 °C. In the primary study, additional saliva samples were collected using OFC swabs (Soma Bioscience, Wallingford, United Kingdom), and s-IgA was quantified immediately using the Cube reader [43].

In the primary study, participants stayed in the same facility and ate the same meals while collecting saliva and urine under identical environmental conditions. Saliva samples and midstream urine samples were collected and transferred to 15 mL Falcon tubes. All participants were women and not menstruating. A total of 6 time-point samples were collected before and after each meal: breakfast (6:30–7:00), lunch (12:00–13:00), and dinner (19:30–20:30). In the primary study, data were collected using a cross-sectional study design at five distinct time points.

#### Appendix A.2.2. Hormone Quantification

Salivary and urinary hormones were measured by enzyme-linked immunosorbent assay (ELISA) and a microplate reader (Varioskan LUX, Thermo Scientific, Waltham, MA, USA). CUBE reader LFD (Soma Bioscience, Wallingford, UK) was also used as an alternative measurement device. Liquid chromatography-time-of-flight mass spectrometry (LC-TOFMS) on an Agilent Technologies 1290 Infinity instrument (Santa Clara, CA, USA) was used to quantify urinary creatinine.

In the ELISA, absolute concentrations were determined by comparing samples across different dilution series; in the LC-TOFMS assay, the detector sensitivity was maintained through prior calibration, and absolute quantification was performed using standards. In both measurements, all samples were measured simultaneously to eliminate batch-related bias.

The preliminary study quantified stress-related hormones, cortisol, Chromogranin A (CgA), and secretory Immunoglobulin A (s-IgA) in saliva samples. These hormones and creatinine in urine samples were quantified. CgA is a protein secreted from the adrenal medulla and sympathetic nerve endings, co-secreted with catecholamines, and reflects the activity of the sympathetic nervous system. Cortisol reflects stress. The s-IgA concentration reflects long-term immunity and short-term stress. The Stress Response Scale-18 (SRS-18) was obtained from five participants. SRS-18 is a self-report questionnaire designed to assess an individual’s psychological and physiological stress responses and to indicate overall stress levels [32,36].

For hormone quantification, samples were thawed at room temperature and then centrifuged at 1500× *g* for 15 min (MDX-310, TOMY, Tokyo, Japan). Commercially available kits of cortisol (1-3002, High-Sensitivity Salivary Cortisol Enzyme Immunoassay (EIA) Kit, Salimetrics, State College, PA, USA), chromogranin A (CgA) (YK070, Human Chromogranin A EIA Kit, Yanaihara Institute, Shizuoka, Japan), salivary s-IgA (1-1602, Salivary Secretory IgA Indirect Enzyme Immunoassay Kit, Salimetrics, State College, PA, USA), urinary s-IgA (KA3980, Secretory IgA Human ELSA Kit, Abnova, Taipei, Taiwan) were used. The kit protocol was followed exactly, except that urine samples were diluted 100 times with diluent for cortisol analysis. Spectrophotometer readings of the samples were taken at 490 nm for CgA and 450 nm for all others using a microplate reader. All samples were tested in duplicates. Quantitative values were calculated using Skanlt Software for Microplate readers (version 7.0.1.4, Thermo Scientific, Waltham, MA, USA).

A 10 μL aliquot of each urine sample was mixed with 90 μL MeOH. Samples were centrifuged at 20,380× *g* for 10 min at 4 °C (MDX-310, Tomy Seiko, Tokyo, Japan), and 90 μL of the supernatant was transferred to a new tube and vacuum dried for 1 h at room temperature (VC-96W; TAITEC, Saitama, Japan). The samples were reconstituted with 10 μL of 90% methanol (*v*/*v*), and 190 μL of H_2_O was added to the sample solution. The sample was diluted 100-fold with H_2_O and then 50-fold with H_2_O containing 0.5 µM internal standard (d6-*N*^1^,*N*^8^-diacetylspermidine). The sample was injected into the LC-MS system. The LC-TOFMS instrument parameters used in this study were as described previously, with slight modifications [44]. This system consists of an autosampler, quaternary pump, and column compartment. MS detection was conducted using an Agilent Technologies G6230B TOF mass spectrometer. In the present study, we used 40 °C as the temperature of the LC columns. The samples were analyzed in positive ion mode.

Data processing was conducted using Agilent MassHunter Qualitative Analysis software (version B.08.00; Agilent Technologies). Peaks of creatinine were extracted based on their retention times and mass-to-charge ratios (*m/z*) corresponding to our standard compounds. The creatinine peak area was divided by the internal standard peak area to obtain a relative peak area, thereby mitigating potential biases arising from MS sensitivity drift. The absolute creatinine concentration in each urine sample was determined from the standard’s relative peak area. The hormone concentration in urine samples was divided by the creatinine concentration.

#### Appendix A.2.3. Data Analysis

In the preliminary study assessing diurnal variations, the nonparametric Friedman test was used for paired data, followed by Dunn’s multiple-comparison test as a post hoc analysis to evaluate differences between the initial sampling time point and subsequent measurements. The correlation between quantitative values obtained with the Cube reader and ELISA was assessed using Spearman’s rank correlation coefficient, and a linear regression analysis was also performed.

### Appendix A.3. Biomarker Results

A preliminary study included the data from five women. Cortisol, CgA, and s-IgA in saliva and urine were quantified (Figure A1). The SRS-18 scores indicated low stress responses across all participants, with one participant scoring 10 and the other four scoring between 0 and 1. It is noteworthy that no participants reported menstruating. Salivary cortisol levels showed significant diurnal variation, with elevated levels before breakfast, followed by a decline and stabilization at lower levels (*p* = 0.0024, Friedman test). Notably, substantial individual variability in salivary cortisol levels was observed before breakfast. Conversely, when urinary cortisol levels were normalized to creatinine, elevated levels were observed around 10:00 AM compared to other time points (*p* = 0.0024). Statistically significant diurnal variations were not detected for CgA in either saliva or urine (*p* = 0.21 and *p* = 0.57, respectively). Salivary s-IgA concentrations tended to be higher before breakfast and lower at other time points (*p* = 0.0029). However, urinary s-IgA concentrations did not demonstrate statistically significant diurnal variations (*p* = 0.31).

Measurement of salivary s-IgA using the CUBE Reader yielded results like those obtained by ELISA, with elevated concentrations before breakfast and diminished concentrations thereafter (*p* = 0.029). A significant positive correlation was observed between s-IgA values obtained by the two methods (R = 0.82, Spearman’s *p* < 0.0001). Linear regression analysis indicated a tendency for the CUBE Reader to yield higher values (Y = 1.3 X + 27). In both methods, the highest values deviated substantially from the regression line.

A preliminary study revealed that salivary cortisol levels remained low and stable after breakfast, exhibiting minimal inter-individual variability. This observation informed the protocol for the main study, which involved collecting saliva samples after breakfast to minimize circadian and inter-individual variability. Furthermore, ELISA was selected for cortisol measurement because elevated cortisol levels are common in high-stress situations. This choice was based on the hypothesis that cortisol levels would be elevated under high-stress conditions.

## Appendix B

**Table A1:** Supplementary Results of Stage-Specific Cross-Sectional Analyses.

Stage	Category	EPDS-	EPDS+	OR	95% CI	*p*-Value
Age group (years)						
1st trimester (pre)	20s	17	3	-	-	-
	30s	30	6	1.13	0.25–5.12	1.0
	40s	2	1	2.83	0.190–42.0	0.45
2nd trimester (pre)	20s	40	1	-	-	-
	30s	56	11	7.86	0.97–63.33	0.028 *
	40s	5	2	16.0	1.22–210	0.052
3rd trimester (pre)	20s	49	5	-	-	-
	30s	93	4	0.422	0.11–1.64	0.21
	40s	8	1	1.23	0.13–11.9	0.86
2 weeks (post)	20s	7	2	-	-	-
	30s	20	2	0.350	0.0412–2.98	0.34
	40s	0	0	-	-	
1 month (post)	20s	12	1	-	-	-
	30s	27	5	2.22	0.234–21.1	0.49
	40s	0	0	-	-	
Gravidity						
1st trimester (pre)	1st	16	3	-	-	-
	2nd	16	3	1.00	0.174–5.72	1.00
	3rd	8	2	1.33	0.184–9.66	1.00
	4th	7	1	0.762	0.0667–8.67	0.83
2nd trimester (pre)	1st	47	6	-	-	-
	2nd	29	8	2.16	0.715–6.52	0.17
	3rd	18	0	0.564	0.110–2.90	0.48
	4th	5	0	0.781	0.0790–7.72	0.83
3rd trimester (pre)	1st	56	2	-	-	-
	2nd	57	5	2.16	0.507–11.9	0.27
	3rd	28	1	0.200	0.115–8.70	1.00
	4th	7	1	0.66	0.174–22.9	0.57
2 weeks (post)	1st	11	2	-	-	-
	2nd	12	1	0.458	0.0547–3.84	0.47
	3rd	4	1	1.38	0.111–17.2	0.80
	4th	2	0	0.917	0.0501–16.8	0.95
1 month (post)	1st	13	3	-	-	-
	2nd	14	2	0.619	0.104–3.69	0.59
	3rd	10	1	0.433	0.0413–4.54	0.48
	4th	3	0	0.619	0.0397–9.64	0.73
History of pregnancy or infant loss						
1st trimester (pre)	No	41	7	-	-	-
	Yes	8	3	2.20	0.466–10.4	0.32
2nd trimester (pre)	No	85	11	-	-	-
	Yes	17	3	1.36	0.343–5.41	0.66
3rd trimester (pre)	No	114	8	-	-	-
	Yes	35	2	0.814	0.165–4.01	0.80
2 weeks (post)	No	25	2	-	-	-
	Yes	4	2	6.25	0.675–57.9	0.11
1 month (post)	No	33	5	-	-	-
	Yes	9	1	0.733	0.0758–7.10	0.79
History of mental health consultation						
1st trimester (pre)	No	41	8	-	-	-
	Yes	8	2	1.28	0.228–7.19	0.78
2nd trimester (pre)	No	91	10	-	-	-
	Yes	11	4	3.31	0.886–12.4	0.075
3rd trimester (pre)	No	137	5	-	-	-
	Yes	13	5	10.5	2.69–41.2	0.0007 ***
2 weeks (post)	No	24	3	-	-	-
	Yes	5	1	1.60	0.137–18.7	0.71
1 month (post)	No	40	5	-	-	-
	Yes	2	1	4.00	0.305–52.5	0.29
Confiding in husband (yes/no).						
1st trimester (pre)	No	0	2	-	-	-
	Yes	48	8	0.0351	0.00154–0.794	0.035 *
2nd trimester (pre)	No	5	4	-	-	-
	Yes	97	10	0.129	0.0332–0.501	0.0028 **
3rd trimester (pre)	No	8	1	-	-	-
	Yes	140	8	0.456	0.0435–4.83	0.51
2 weeks (post)	No	3	0	-	-	-
	Yes	26	4	1.00	0.0417–24.0	1.0
1 month (post)	No	3	1	-	-	-
	Yes	39	4	0.308	0.0272–3.47	0.35
Ability to confide in biological mother						
1st trimester (pre)	No	4	2	-	-	-
	Yes	40	7	0.350	0.0515–2.38	0.28
2nd trimester (pre)	No	7	2	-	-	-
	Yes	89	12	0.472	0.100–2.22	0.34
3rd trimester (pre)	No	13	6	-	-	-
	Yes	126	2	0.0339	0.00621–0.185	<0.001 ***
2 weeks (post)	No	5	0	-	-	-
	Yes	23	4	1.00	0.0408–24.5	1.0
1 month (post)	No	4	1	-	-	-
	Yes	37	5	0.541	0.0529–5.52	0.61
Availability of other consultation support						
1st trimester (pre)	No	3	1	-	-	-
	Yes	46	9	0.588	0.0541–6.41	0.66
2nd trimester (pre)	No	5	4	-	-	-
	Yes	97	10	0.129	0.0332–0.501	0.0028 **
3rd trimester (pre)	No	5	2	-	-	-
	Yes	145	8	0.138	0.0261–0.729	0.020 *
2 weeks (post)	No	1	0	-	-	-
	Yes	28	4	1.00	0.0370–27.0	1.0
1 month (post)	No	4	1	-	-	-
	Yes	38	5	0.526	0.0515–5.41	0.59
Perceived economic difficulty						
1st trimester (pre)	No	35	7	-	-	-
	Yes	13	3	1.15	0.297–4.47	0.84
2nd trimester (pre)	No	84	9	-	-	-
	Yes	18	4	2.07	0.553–7.77	0.28
3rd trimester (pre)	No	131	5	-	-	-
	Yes	19	5	6.92	1.92–25.0	0.0031 **
2 weeks (post)	No	24	4	-	-	-
	Yes	5	0	0.462	0.0252–8.47	0.60
1 month (post)	No	37	5	-	-	-
	Yes	5	1	1.37	0.159–11.8	0.77
Satisfaction with residential environment for child-rearing						
1st trimester (pre)	No	14	5	-	-	-
	Yes	35	5	0.40	0.100–1.60	0.20
2nd trimester (pre)	No	13	7	-	-	-
	Yes	89	7	0.146	0.0441–0.484	0.0017
3rd trimester (pre)	No	17	4	-	-	-
	Yes	133	6	0.192	0.0491–0.749	0.017
2 weeks (post)	No	7	3	-	-	-
	Yes	22	1	0.106	0.00945–1.19	0.069
1 month (post)	No	6	1	-	-	-
	Yes	36	5	0.833	0.0823–8.43	0.88
Attachment styles (four-category model)						
1st trimester (pre)	Secure	24	5	-	-	-
	Dismissing	7	0	-	-	0.56
	Preoccupied	4	2	2.40	0.341–16.9	0.58
	Fearful	14	3	1.03	0.213–4.97	1.0
2nd trimester (pre)	Secure	52	4	-	-	-
	Dismissing	7	1	1.86	0.181–19.1	0.50
	Preoccupied	16	4	3.57	0.729–14.5	0.20
	Fearful	24	5	2.71	0.667–11.0	0.26
3rd trimester (pre)	Secure	87	0	-	-	-
	Dismissing	13	0	-	-	1.0
	Preoccupied	16	3	-	-	0.0050 **
	Fearful	31	6	-	-	<0.001 ***
2 weeks (post)	Secure	17	0	-	-	-
	Dismissing	1	1	-	-	0.11
	Preoccupied	5	1	-	-	0.26
	Fearful	7	2	-	-	0.11
1 month (post)	Secure	29	1	-	-	-
	Dismissing	3	2	19.3	1.33–282	0.047 *
	Preoccupied	4	2	14.5	1.06–199	0.066
	Fearful	5	1	5.8	0.310–109	0.31
Attachment security (two-category model)						
1st trimester (pre)	Secure	24	5	-	-	-
	Insecure	25	5	1.04	0.270–4.06	0.96
2nd trimester (pre)	Secure	52	4	-	-	-
	Insecure	47	10	2.50	0.740–8.43	0.14
3rd trimester (pre)	Secure	87	0	-	-	-
	Insecure	60	9	-	-	-
2 weeks (post)	Secure	17	0	-	-	-
	Insecure	13	4	7.36	0.358–151	0.19
1 month (post)	Secure	29	1	-	-	-
	Insecure	12	5	12.1	1.35–109	0.026 *
SRS-18						
1st trimester (pre)	Low	43	4	-	-	-
	High	6	6	10.8	2.34–49.5	0.0023 **
2nd trimester (pre)	Low	94	6	-	-	-
	High	6	8	20.9	5.46–80.0	<0.001 ***
3rd trimester (pre)	Low	139	6	-	-	-
	High	6	4	15.4	3.42–69.6	<0.001 ***
2 weeks (post)	Low	27	2	-	-	-
	High	1	2	27.0	1.65–443	0.035 *
1 month (post)	Low	39	3	-	-	-
	High	2	3	19.5	2.29–166	0.011 *

Stage indicates the timing relative to childbirth, where “pre” refers to the prenatal period and “post” refers to the postpartum period. For all categorical variables, the first (top) row represents the reference category. Odds ratios (ORs) and 95% confidence intervals (CIs) were not estimated when any cell count was zero (no continuity correction was applied). *p* values were calculated using Fisher’s exact test (two-sided). These analyses were exploratory. * *p* < 0.05, ** *p* < 0.01, and *** *p* < 0.001.
